# Is Photosynthetic Capacity Associated With Diversification of C_3_ Plants?

**DOI:** 10.1111/ele.70331

**Published:** 2026-02-10

**Authors:** Christopher D. Muir

**Affiliations:** ^1^ Department of Botany University of Wisconsin Madison Wisconsin USA

**Keywords:** ancestral state reconstruction, diversification, macroevolution, photosynthesis, phylogenetic comparative methods

## Abstract

Schweiger and Schweiger (2024) recently proposed that the evolution of greater photosynthetic capacity in C_3_ plant species may be linked to increased species diversification. This conclusion is premature because their methods cannot reliably estimate diversification rates or ancestral trait values. Reanalyzing the data set using model‐based phylogenetic comparative methods reveals no evidence that photosynthetic capacity is associated with diversification rates among C_3_ plant genera.

## Graphical abstract

Schweiger and Schweiger (2024) linked photosynthetic capacity to diversification rate in C_3_ vascular plants. However, the diversification rate estimates were not conditioned on extinction or incomplete sampling, and the ancestral trait reconstruction was based on unrealistic assumptions that are highly sensitive to incomplete sampling. Reanalysis using model‐based phylogenetic comparative methods (PCMs) in this paper finds no association between photosynthetic capacity and diversification rate among vascular plant genera.

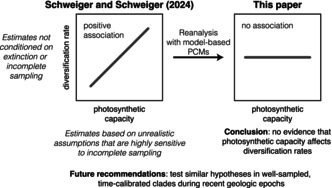

Phylogenetic comparative methods (PCMs) are widely used to infer historical patterns of species diversification and phenotypic evolution based on the relationships among extant species and their trait values (Pennell and Harmon 2013). Recently Schweiger and Schweiger (2024) purport to show that greater photosynthetic capacity is linked to greater diversification among C_3_ vascular land plants over the past 80 million years. Specifically, they argue that diversification rates increased along with photosynthetic capacity ($V_{\text{cmax}}$ and $J_{\max}$) as atmospheric CO_2_ decreased throughout the Miocene. This conclusion is premature because the PCMs Schweiger and Schweiger (2024) use to estimate diversification rates and ancestral states do not account for and are highly sensitive to incomplete taxon sampling.

## 1 Diversification Analysis

Diversification rates are typically inferred using the distribution of branch lengths in a molecular phylogeny. The reconstructed evolutionary process, even with complete sampling of extant taxa and perfect information, cannot infer the presence of lineages that left no descendants because of extinction (Nee, May, and Harvey 1994). Incomplete taxon sampling acts like a recent extinction event because nodes leading to unsampled taxa cannot be inferred. Valid inference of diversification rates must therefore condition on both extinction and incomplete sampling. Schweiger and Schweiger (2024) analyse a molecular phylogeny of vascular land plants containing 232 species, 0.065% of the 359,208 currently accepted species as of December 2024 (Kindt 2020). They estimate diversification rate as $\log\left(n\right)/t$, where n is the number of tips and t is the stem group age (Magallon and Sanderson 2001). Since apparent n is a small fraction of its true value, this statistic cannot estimate diversification rate. The Kendall‐Moran estimate of speciation rate used by Schweiger and Schweiger (2024) works for completely sampled phylogenies with no extinction (Baldwin and Sanderson 1998), an untenable assumption in this case. Schweiger and Schweiger (2024) compound these errors by pruning tips and branches younger than each time interval for which they estimate diversification rates. Thus, at each interval they artificially remove nodes in the phylogeny, further biasing downward estimates of diversification.

Random incomplete sampling biases downward estimates of diversification rates in the recent past (Nee, Holmes, et al. 1994). The bias caused by random incomplete sampling is opposite the pattern Schweiger and Schweiger (2024) observe of more rapid diversification toward the present. Apparent increases in diversification in younger clades are a general phenomenon not confined to C_3_ plants (Harmon et al. 2021) that may be a result of bias toward sampling species‐rich clades (Louca et al. 2022). Reliable inference must address both incomplete and nonrandom sampling.

## 2 Ancestral Trait Analysis

**FIGURE 1:**
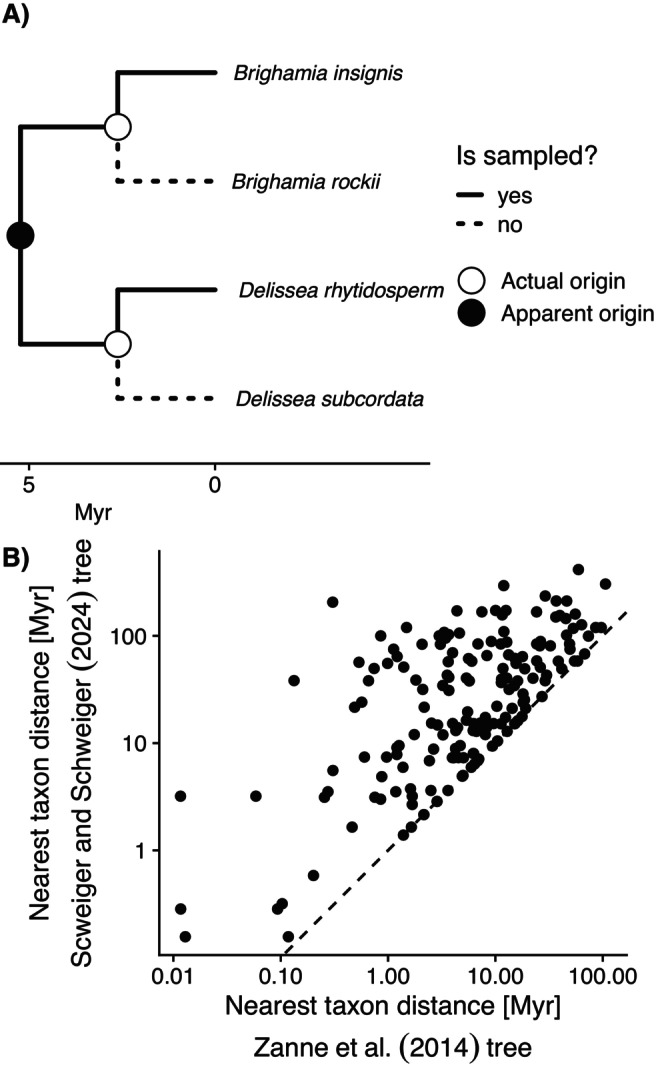
The method Schweiger and Schweiger (2024) use to infer when a species originated is misleading when taxon sampling is incomplete. (A) For example, the Hawaiian lobeliads *Brighamia insignis* A. Grey and *Delissea rhytidosperma* H. Mann in their data set are inferred by this method to have “originated” 5.23 mya, as shown in the phylogeny above. This apparent origin (black point on more ancient node) is an artefact of more closely related congeneric species ( *B. rockii* H. St. John and *D. subcordata* Gaudich.) not being sampled (dashed branches leading to unsampled species). The node of the apparent origin represents the most recent common ancestor of these species, but both species actually diverged from more closely related species much more recently (white points). The timing of divergence from putative sister species is approximate and for illustration purposes only (based on Givnish et al. 2009). (B) The consequences of this methodological choice are that the divergence times used by Schweiger and Schweiger (2024) (y‐axis) are biased upward by an average of 315% and modestly correlated ($r^{2}=0.43$ on log–log scale) with the divergence times estimated from the full phylogeny (x‐axis) from Zanne et al. (2014). Those divergence times are themselves only loosely related to the true divergence time between sister species because of mostly incomplete sampling even in the unpruned Zanne et al. (2014) phylogeny.

Unobserved ancestral states must be reconstructed using a probabilistic model of trait evolution, but inferred states are often impossible to independently verify (but see Polly 2001). There is a high likelihood that estimates from misspecified models are misleading (Revell 2025). Schweiger and Schweiger (2024) do not estimate ancestral states based on a trait model, but instead calculate it as the “median trait values from all species that originated in the respective time period,” where time period refers to a 5‐million‐year interval. There are two fundamental errors with this approach. First, because the phylogeny contains a small fraction of all species, the origin time of a species (when it split from its sister species) will be biased upward by an unpredictable amount (Figure 1a). The nodes used by Schweiger and Schweiger (2024) are on average 315% older than and modestly correlated with (log–log $r^{2}=0.43$) the nodes separating nearest species in the unpruned Zanne et al. (2014) tree (Figure 1b), which itself contains 31,389 species, representing only 8.7% of described vascular land plant species.

**FIGURE 2:**
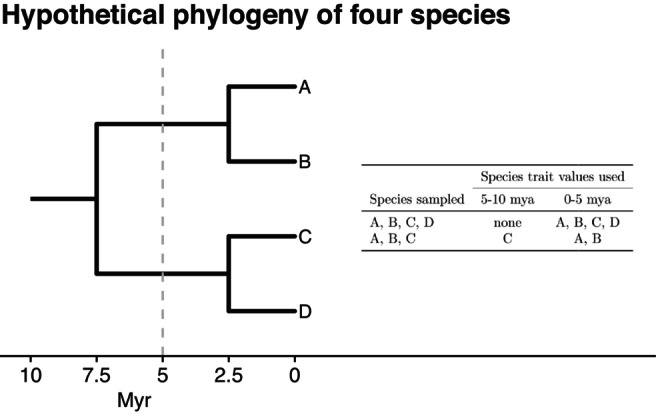
The method Schweiger and Schweiger (2024) use to estimate ancestral trait values is sensitive to small differences in taxon sampling. Assume we know the true phylogeny of four species A, B, C, and D (left). The species A and B are sister species that split 2.5 mya, likewise for C and D. The common ancestor of all four split 7.5 mya. If we sampled all four species, their trait values would contribute to the estimated average trait value in the interval between 0 and 5 mya. This clade would not contribute to the estimated average trait value in the interval between 5 and 10 mya because the species did not originate in that interval. However, it is clear that the ancestors of these species must have existed during that interval. Now consider the effect of incomplete sampling. If species D were not sampled, then the trait values for A and B would contribute to the 0–5 mya interval and species C would contribute to the 5–10 mya interval. The species used to calculate average trait values at different time intervals changes depending on whether all species are sampled or a subset are sampled (right), even though the underlying biological reality is unchanged.

Second, the method for estimating average trait values in past intervals implies strong and unrealistic assumptions about trait evolution, is inappropriate for a sparsely sampled phylogeny, and is highly sensitive to small differences in taxon sampling. Even if species' origination dates were inferred correctly, estimating ancestral states by assuming they are equal to the state of the extant descendant implies all trait evolution occurs during speciation events (cladogenic) and not along lineages (anagenic). Assuming cladogenic evolution is especially problematic in this case because most nodes in the phylogeny are not sampled, thereby missing most cladogenic events when trait change would occur by their implied assumptions. A median of 7 nodes per species were pruned from the Zanne et al. (2014) phylogeny in their analysis, but this is a severe undercount of missing nodes because of both incomplete sampling in the unpruned tree and extinction. Finally, estimates of past trait values using this method are highly sensitive to small changes in taxon sampling. A minor change in sampling can result in quite different estimates even with the same underlying evolutionary scenario (see Figure 2 for a worked example).

## 3 No Evidence That Photosynthetic Capacity Is Linked to Diversification in Seed Plants

**FIGURE 3:**
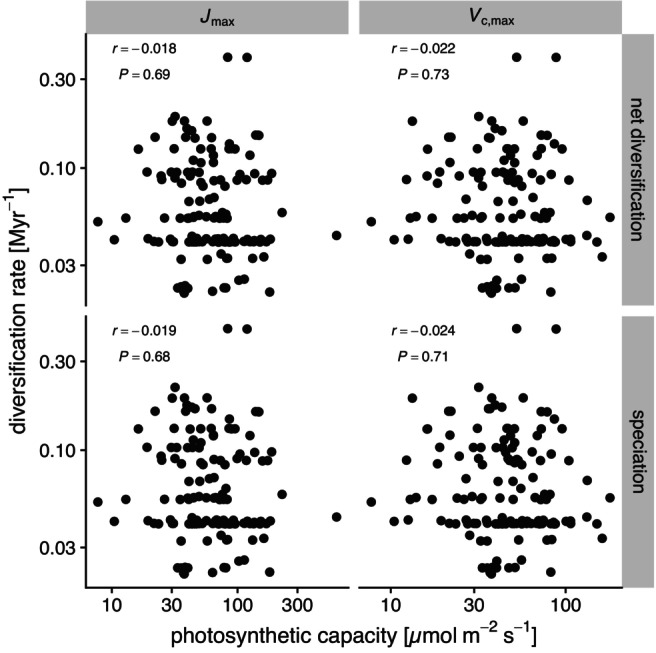
No evidence that photosynthetic capacity explains variation in speciation or net diversification rates among C_3_ seed plant genera. Net diversification (top row) and speciation (bottom row) rates estimated using BAMM version 2.5.0 on a nearly‐complete phylogeny of seed plant genera (Dimitrov et al. 2023) are not significantly associated with average $J_{\max}$ (left column) or $V_{\text{cmax}}$ (right column) based on STRAPP permutation tests (Rabosky and Huang 2016). Both axes are log‐scaled. The estimated Pearson product moment correlation r and permutation based *p*‐values from two‐tailed tests are in the upper‐left of each facet.

Several PCMs have been developed in the past decade to estimate whether diversification rates vary among lineages and through time in association with traits (Martínez‐Gómez et al. 2024; Morlon et al. 2024). These methods account for extinction and incomplete sampling and therefore can, in principle, test whether diversification is associated with traits like $V_{\text{cmax}}$ or $J_{\max}$. When extinction and incomplete sampling are accounted for using BAMM version 2.5.0 (Rabosky 2014) on a nearly‐complete phylogeny of seed plant genera (Dimitrov et al. 2023), there is no evidence that speciation or net diversification rates are associated with $V_{\text{cmax}}$ or $J_{\max}$ (Figure 3) based on permutation tests developed by Rabosky and Huang (2016) (see Appendix A). This method answers a slightly different question than that posed by Schweiger and Schweiger (2024) because it tests for an association between tip rates and traits, not ancestral rates and traits. However, the tip rates of many genera encompass much of the Miocene when global CO_2_ declined and tip rates suffer from fewer identifiability issues (Louca and Pennell 2020). Alternative methods not considered here are CLaDS2 (Barido‐Sottani and Morlon 2023) and QuaSSE (FitzJohn 2010). While these PCMs account for extinction and incomplete sampling, no method performs well when taxon sampling is extremely sparse, samples are biased, or when making inferences about the deep past from extant taxa. Certain questions may be better suited to specific clades with (near‐)complete sampling (Donoghue and Edwards 2019), well resolved and fossil‐calibrated nodes, and to relatively recent geological epochs.

## Author Contributions

C.D.M. conceived of the ideas and wrote the manuscript.

## Funding

This work was supported by the Division of Environmental Biology, 2131817.

## Conflicts of Interest

The author declares no conflicts of interest.

## Data Availability

Data are available from previously published sources. The code to reproduce analyses is available on GitHub (https://github.com/cdmuir/photosynthesis‐diversification) and archived on Zenodo (https://doi.org/10.5281/zenodo.17835088).

## Appendix A Estimating the Relationshp Between Photosynthetic Capacity and Diversification Rates Using BAMM

The speciation and net diversification rates estimated by Dimitrov et al. (2023) were unavailable from the authors, but I reanalyzed their molecular phylogeny of seed plant genera using their sampling fractions and following the same procedures in BAMM as described in their paper. For the STRAPP analysis, I estimated the correlation between log‐transformed rates and traits using 1000 permutations to generate the null distribution of the Pearson product–moment correlation. The $V_{c,\max}$ and $J_{\max}$ trait data from Schweiger and Schweiger (2024) were averaged at the genus level when more than one species per genus was present. This was necessary because Dimitrov et al. (2023) estimated a single diversification rate per genus. Although this method uses macroevolutionary models to account for extinction and incomplete sampling, the results should be interpreted with extreme caution. For example, it does not account for uncertainty in the phylogeny, the species sampling is extremely sparse, variation within genera is ignored, and temporal variation in diversification rates may not be identifiable from extant taxa only (Louca and Pennell 2020).
